# Artificial intelligence, machine learning and health systems

**DOI:** 10.7189/jogh.08.020303

**Published:** 2018-12

**Authors:** Trishan Panch, Peter Szolovits, Rifat Atun

**Affiliations:** 1 Wellframe Inc., Boston, Massachusetts, USA; 2 Department of Electrical Engineering and Computer Science, Computer Science and Artificial Intelligence Laboratory, Massachussetts Institute of Technology, Cambridge, Massachusetts, USA; 3 Department of Global Health and Population, Harvard TH Chan School of Public Health, Harvard University, Boston, Massachusetts, USA; 4 Department of Global Health and Social Medicine, Harvard Medical School, Harvard University, Boston, Massachusetts, USA

Globally, health systems face multiple challenges: rising burden of illness, multimorbidity and disability driven by ageing and epidemiological transition, greater demand for health services, higher societal expectations and increasing health expenditures [1]. A further challenge relates to inefficiency, with poor productivity [2]. These health system challenges exist against a background of fiscal conservatism, with misplaced economic austerity policies that are constraining investment in health systems.

Fundamental transformation of health systems is critical to overcome these challenges and to achieve universal health coverage (UHC) by 2030. Machine learning, the most tangible manifestation of artificial intelligence (AI) – and the newest growth area in digital technology – holds the promise of achieving more with less, and could be the catalyst for such a transformation [3]. But the nature and extent of this promise has not been systematically assessed.

To date, the impact of digital technology on health systems has been equivocal [4]. Is AI the ingredient for such a transformation, or will it face the same fate as earlier attempts at introducing digital technology? In this paper, we explore potential applications of AI in health systems and the ways in which AI could transform health systems to achieve UHC by improving efficiency, effectiveness, equity and responsiveness of public health and health care services.

## EVOLUTION OF AI AND MACHINE LEARNING

AI is a broad discipline that aims to understand and design systems that display properties of intelligence (**Box 1**) – emblematic of which is the ability to learn: to derive knowledge from data. This is a broad definition that arguably has some cross over with existing statistical techniques [6]. The recent explosion in progress in this field is attributable to a subset of AI – machine learning and one family of techniques in particular, deep learning, where computers are programmed to learn associations based on large quantities of raw data such as the pixels of digital images. Deep learning systems have been applied extensively and set new benchmarks in areas of the economy where high quality digital data are plentiful and there is a strong economic incentive to automate prediction tasks [5].

Box 1 **Evolution of Artificial Intelligence and Machine Learning.**

**Artificial Intelligence (AI)**

A broad scientific discipline with its roots in philosophy, mathematics and computer science that aims to understand and develop systems that display properties of intelligence.

**Machine Learning**

A sub discipline of AI, where computers programs (algorithms) learn associations of predictive power from examples in data. Machine learning is most simply the application of statistical models to data using computers. Machine learning uses a broader set of statistical techniques than those typically used in medicine. Newer techniques such as Deep Learning are based on models with less assumptions about the underlying data and are therefore able to handle more complex data.

**Deep Learning**

Deep learning methods allow a machine to be fed with large quantities of raw data and to discover the representations necessary for detection or classification. Deep learning methods rely on multiple layers of representation of the data with successive transformations that amplify aspects of the input that are important for discrimination and suppress irrelevant variations . Deep learning may be supervised or unsupervised. Deep learning methods have been responsible for many of the recent foundational advances in machine learning [5].

**Supervised Learning**

Training computer programs to learn associations between inputs and outputs in data through analysis of outputs of interest defined by a (typically human) supervisor. Once associations have been learned based on existing data they can be used to predict future examples. This is one of the most established areas of machine learning with multiple examples inside and outside health care.

**Unsupervised Learning**

Computer programs that learn associations in data without external definition of associations of interest. Unsupervised learning is able to identify previously undiscovered predictors, as opposed to simply relying on known associations (s30).

**Reinforcement Learning**

Computer programs that learn actions based on their ability to maximize a defined reward. This approach is influenced by behavioural psychology and has been applied with considerable success in gaming where there is perfect information, many possible options and no real world cost of failure.

In these cases, deep learning algorithms have been able to uncover associations of predictive value, typically for a single use case, with large amounts of data and human expertise to curate the data and tune the algorithms involved [7]. These advances in machine learning are not a prototype for “artificial general intelligence”: a broad general-purpose intelligence that can, like the human brain, independently be deployed across use cases and independently incorporate learned concepts together in a self-reinforcing cycle.

## AI AND DECISION MAKING IN HEALTH SYSTEMS

Effective management of health systems, like the provision of public health or health care, is in essence a lattice of information processing tasks. Policy makers modify health system functions of organisation and governance, financing and resource management to achieve health system outputs (health care services and public health) and system goals [8],.

The provision of health care itself involves two core information processing tasks: first, screening and diagnosis, which is the classification of cases based on history, examination and investigations, and second treatment and monitoring, which involves the planning, implementation and monitoring of a multistep process to deliver a future outcome.

The essential form of these processes across the domains of health system management and the provision of care involve hypothesis generation, hypothesis testing and action. Machine learning has the potential to improve hypothesis generation and hypothesis testing tasks within a health system by revealing previously hidden trends in data, and thus has the potential for substantial impact both at the individual patient and system level.

Machine learning expands on existing statistical techniques [6], utilising methods that are not based on a priori assumptions about the distribution of the data, and can find patterns in the data that can in turn be used to formulate hypotheses and hypothesis tests. Thus, whilst machine learning models are more difficult to interpret, they can incorporate many more variables and are generalizable across a much broader array of data types, and can produce results in more complex situations [9]. These methods have been deployed in the research context in screening and diagnosis and prediction of future events (**Table 1**). These deployments are in disparate areas, typically in hospital rather than community setting, and in the vast majority of cases based on data from single centers, with implications for reproducibility [11] and generalizability [12]. However, the rapid pace of development of machine learning continues both within health care and more broadly across all information processing tasks in society [13].

**Table 1:** Applications of artificial intelligence in diagnosis and prediction

Diagnosis and case identification	Prognosis and prediction
**Waveform analysis:**	
Obstetrics – intrapartum monitoring (s31)	Cardiovascular risk prediction (s32-34)
Neurology – remote monitoring of gait (s35)	Prediction of breast cancer survival (s36)
**Image analysis:**	
Pathology – detection of lymph node metastases in breast cancer (s37)	Prediction of outcomes in colorectal cancer (s38)
Dermatology – identification of benign and malignant tumors (s39), identification of fungal infection (s40), classification of skin cancer (s41)	Predicting of survival in non-small cell lung cancer (s42)
Ophthalmology – identification of diabetic retinopathy (s43), grading of macular degeneration (s44)	Prediction of hospitalization due to heart disease (s45)
Cardiology – diagnosis of acute coronary syndrome (s46), identification of heart failure status through remote patient monitoring (s47)	Prediction of primary care utilization (s48)
Radiology – mammography (s49), diagnosis of pneumonia from chest x-ray [10]	Prediction of sepsis in the intensive care unit, emergency department and on the hospital floor (s50)
**Electronic health record analysis:**	Prediction of central line associated infections and mortality (s51)
Prediction of inpatient diagnosis (s52); Identification of sepsis in the emergency department (s53); Identification of breast cancer symptoms (s54); Heart failure case identification (s55); Identification of patient phenotype from analysis of ICU data (s56); Identification of medical subdomains in clinical notes (s57); Extraction of ICD-10 codes from death certificates and autopsy reports (s58)	Prediction of treatment outcome in social anxiety (s59)
**Claims analysis:**	Prediction of psychiatric readmission from discharge summaries (s60)
Screening for type 2 diabetes mellitus from payer claims data (s61]	

*References s21 to s61 are available in the **Online Supplementary Document(Online Supplementary Document)**.

## POTENTIAL EFFECT OF AI ON CLINICAL CARE AND HEALTH WORKFORCE

Machine learning has become a “General Purpose Technology”, in that it is pervasive, can be improved over time and has the potential to spawn complementary innovations [14]. The implementation of such technologies tends to result in “widespread economic disruption, with concomitant winners and losers” [15]. Economists Acemoglu and Restrepo, who studied the historical effects of automation – the process of substitution of mechanization for human labour – argue that automation exerts a *displacement effect* where human labour is displaced by machines in areas where machines have differential advantage [16]. However, countervailing forces that increase demand for labour offset this displacement effect: a *productivity effect,* as operations become more efficient and less costly. This in turn allows savings to be invested on existing non-automatable tasks and on the creation of new non-automatable tasks, some of which involve directly working on the automating technology.

To see how this general trend might apply to the health care workforce it is useful to examine the clinical area that is currently best represented in machine learning literature, diagnostic radiology.

As deep learning algorithms have set new performance benchmarks in diagnostic image analysis, some commentators have forecast the inevitable demise of radiologists and questioned the need for training new radiologists [17]. It is plausible that machine learning will enable existing radiologists to handle more cases and then, as machine learning systems are able to work more autonomously, to transfer responsibility for diagnostic image analysis to non-radiologists supported by machine learning systems. Such reorientation of tasks would create an opportunity for health systems to recalibrate the skill mix of radiology teams and their distribution, with more tasks done at the primary care level and non-automatable work and rarer cases handled by a smaller number of radiologists at secondary and tertiary centers.

The researchers behind a machine learning system responsible for pneumonia diagnosis [18] have developed a tool where the technology system “reads” the image first and highlights areas for the human radiologist to focus on – thereby improving workflow efficiency by allowing a human decision maker to focus her limited attention where it can be most effectively deployed and deal with many more cases [10]. One would expect the same applications to also transform pathology and other specialties reliant upon image analysis [19,20].

Machine learning will thus create processes performed by a hybrid of *human and computer*. These instances offer the potential to achieve optimal combination of leveraging human ability to generate hypotheses, collaborate and oversee AI systems to harness AI ability to analyse large volumes of data to find associations with predictive power or optimise against a success criterion. Jha and Topol propose that radiology and pathology should be amalgamated into a new specialty called an “information specialist”, whose responsibility will not be so much to extract information from images and histology but to manage the information extracted by artificial intelligence in the clinical context of the patient (reference s21 in **Online Supplementary Document(Online Supplementary Document)**). It is plausible that the quality and scope of care will increase considerably whilst costs may stay relatively constant unless other specialties can be amalgamated in this way and more work shifted to primary health care.

**Figure:**
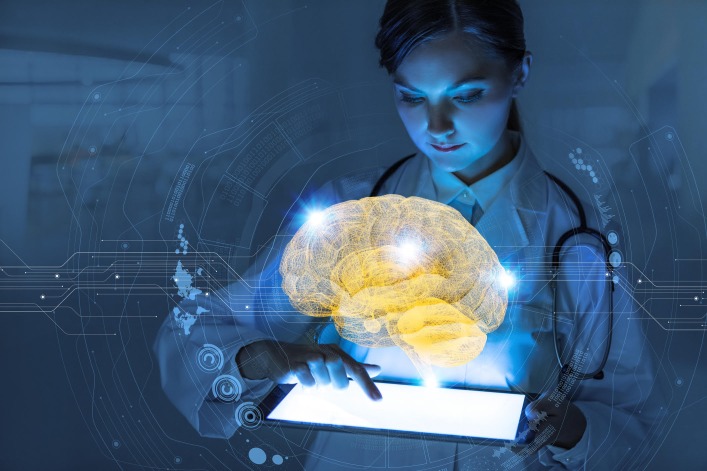
Photo: iStock

## CREATING A RECEPTIVE CONTEXT FOR HARNESSING THE BENEFITS OF AI IN HEALTH SYSTEMS

Machine learning is a rapidly advancing technology. Whilst there are significant technical leaps forward to come, as for any new technology, it will not just be technical challenges that limit the application of machine learning in health, but rather the absence of a receptive context for their adoption and diffusion (s22,s23).

A receptive context for AI requires, among others, availability of curated data, an enabling regulatory environment, legal provisions to safeguard citizens’ rights, clear rules on accountability, and capacity to manage strategic change to enable appropriate introduction and application of machine learning.

### Curation of data

A perennial problem in health information technology is interoperability – the absence of a common data schema for health care organisations has made it difficult to combine data across a health system. Current approaches (s24) in health systems do not support existing data aggregation needs and should be reconsidered to support the additional data requirements of machine learning.

However, a recent unpublished study from Google and three academic medical centers demonstrated how data could be combined across three teaching hospitals without translation into a common format first. In this study deep learning was applied to data in its native format, without transformation into a common data structure first, and produced results exceeding existing performance benchmarks in predicting in-hospital mortality, 30-day unplanned readmission, prolonged length of stay and all of a patient’s final diagnoses (s25). Whilst advances in machine learning, such as this example, may reduce the need for data harmonization across a health system, they do not eliminate it. It is still important that health systems support initiatives to aggregate data both for existing functions and to support machine learning. In fact, the potential impact of machine learning should redouble motivation behind these initiatives.

It is likely that machine learning will deliver technically superior performance, but it will not be perfect. If successful, many people will benefit, but undoubtedly some will also be worse off. It is plausible that those negatively affected will be from marginalized groups who might be underrepresented in the data sets used to build machine learning algorithms. As such, while machine learning may deliver superior technical performance, it could compound inequities. To tackle the risk of further widening inequities, it is essential that there is adequate diversity of individuals represented in data sets, data are used from different clinical sites, and diversity is present in those developing machine learning algorithms. Without these provisions, examples of “algorithmic bias” (s26) and reinforcement of social inequities demonstrated elsewhere in society will be demonstrated in health care applications.

### Trust and data management

An important barrier to the creation of data sets necessary for the development of machine learning systems is the lack of trust regarding how data will be used. Recent examples of over-zealous data sharing, where data have been shared transgressing legislative safeguards, have further eroded trust betwee citizens whose data are used and those using the data (s27).

Whilst in the consumer digital economy it is broadly accepted that as citizens we offer our data in return for better search results or a more relevant social network feed, for patients it is not clear that the same implicit understanding of the value of this quid pro quo is present. This could be because patients have simply not witnessed the benefits of data sharing in the way that retail consumers have. Further, retail and health sectors are qualitatively different, and the core concerns regarding trust in governments, privacy or fears regarding discrimination based on health status loom larger in patients’ minds than any potential distal benefits of data sharing.

### Working with the technology industry

The inconvenient truth is that advances in machine learning are going to originate from or require co-operation with a handful of technology companies that have already invested billions of dollars to aggregate the intellectual capital and necessary computing and storage resources for machine learning. However such concentration accentuates broader concentration in the economy and introduces additional legislative complexity for national governments, which could end up being reliant on a handful of private technology companies for core infrastructure for AI.

As such, novel contracting mechanisms are necessary to work with these private technology companies to enable capture and use of health data at a national scale whilst maintaining privacy and fair attribution of intellectual property created. No such agreement is in place currently and there is no consensus about how to develop one. The absence of an agreed framework for contracting and intellectual property rights provides a significant opportunity for international public health organisations to display leadership and for the private corporate entities to demonstrate corporate social responsibility and to safeguard social solidarity to ensure the benefits of AI are broadly shared.

A broader conversation is needed between citizens, curators of data in health systems and private companies involved in AI and machine learning in health care. This conversation should seek to resolve issues pertaining to intellectual property related to health data, and the trade off between health data as a public good and as private capital and address patient concerns regarding privacy.

### Accountability

Machine learning systems, in particular deep neural nets, are effectively ‘black boxes’ – their operations involve millions of data points used to calibrate models that can generate thousands of classifications – where the byzantine inner representation of said data are not typically intelligible to a human observer. As such, the internal process of generating an inference from data are not describable in the same way as traditional statistical models are.

The European Union is in the process of enforcing new General Data Protection Regulation which will effectively create a “right to explanation,” where an individual has the right to request an explanation of a decision that was made about them using “automated processing” (s28). This will generate challenges for decisions where processes generating them cannot be clearly explained.

In a recent paper on “Accountability of AI Under the Law”, Harvard University’s Berkman Klein working group on Explanation and the Law analyse the issue of accountability of machine learning systems (s29). They define mechanisms for accountability that are dependent on the type of problem being addressed by the machine learning system. For more defined problems theoretical constraints or statistical evidence from trials of machine learning systems might be sufficient, but in the type of problems that are experienced in clinical practice, where the objectives are not always clear and there is a high likelihood of external factors, *explanation* is necessary for accountability. Explanation is defined as permitting “an observer to determine the extent to which a particular input was determinative or influential to an output.” They recommend that an AI system should be expected to provide an explanation in situations where a human decision maker would be expected to do the same. In order to achieve this, however, technology investments are necessary to create distinct explanation systems that are able to communicate the inner workings of machine learning (in particular deep learning) algorithms, assuming that this is possible to do.

### Capacity for managing strategic change

For machine learning based diagnosis, care management and monitoring to be adopted in practice, demonstration of algorithmic superiority alone will not be sufficient. To convince clinicians and policy makers, machine learning enabled systems will have to deliver outcomes of interest in practice through experimental trials or through real world observations of performance. Machine learning, however, is a moving target, and such initiatives may need to be repeated as algorithms improve with greater availability of data and better techniques. This may incur a significant cost to health systems, which will need to offset these costs by improvements in performance and health workforce efficiency.

The uncertainty around how machine learning will impact on the workforce – within health care and more broadly – is a concern to policy makers. Most likely, the impact of the aforementioned ‘displacement effect’ will be felt most acutely by those in lower skilled manual and non-manual occupations. In health systems with currently adequate numbers of health workforce, this displacement may generate a larger pool of workers seeking employment, particularly those involving psychological and emotional well-being and caring for the elderly and disabled – typically occupations that are considered skilled and non-automatable. With potentially a greater supply of front line care workers and machine learning systems, there is an opportunity for improved chronic disease management and community based care for ageing populations. However, this may not be sufficient to counteract the broader effects of automation on labour markets, and governments will need to make proactive investments in retraining workers displaced to prepare them for alternative opportunities, including new roles in the development and curation of data sets and machine learning algorithms.

Conversely, in low- and middle-income countries where there is an acute shortage of health workforce, machine learning offers the real opportunity to expand health care service coverage and increase the likelihood of achieving universal health coverage.

## CONCLUSIONS

In this paper we have discussed the direct impact of machine learning on health systems, but have not explored the indirect effects of machine learning in basic sciences, drug discovery and other enabling technologies on health systems.

Prediction is inherently difficult: technology modifies its environment and the environment then generates further opportunities and new constraints for the technology. Ultimately, general purpose intelligence will be possible, as a version of it already exists in human brains. However, an extrapolation of existing techniques to re-create general intelligence artificially appears unlikely in the next 5-10 years.

However, what is immediately plausible, and should therefore be planned for, is a federation of ‘narrow’ and ‘targeted’ machine learning systems that are able to tackle core information processing problems across a health system by augmenting capabilities of human decision makers, and in so doing establishing new standards of effectiveness and efficiency in clinical and management operations. This is a significant opportunity for health system transformation as the cost of augmenting decision-making capabilities across the health system is unlikely to be large. There is no other approach that offers such potential impact without commensurate scaling of cost. The fixed cost involved in developing machine learning solutions: the cost of research and development and of re-tooling a health system is considerable, but given the potential scalability, the rationale to invest is clear.

An opportunity exists to seed growth in machine learning through the creation of high resolution clinical data sets and the necessary mechanisms for sharing of data and collaborative investigation to establish both efficacy and safety. What is currently missing in health systems is the leadership to do so. Whilst the issues raised are being actively discussed among the academic AI community, the academic AI community alone will not be able to solve them – it will require leadership from policy makers and the engagement of citizens, patients and clinicians. The fear of wholesale displacement of health workforce by AI is overstated, but where fear is warranted is in considering the opportunity cost of not embracing AI, of continuing business as usual with piecemeal implementation of AI that does not realize its potential for transformation of health systems.
