# Supplementary material for: Artificial intelligence, machine learning and health systems
Source: J Glob Health. 2018 Oct 21;8(2):020303. doi: 10.7189/jogh.08.020303 (PMC6199467; doi:10.7189/jogh.08.020303)
Supplement: Online Supplementary Document [file jogh-08-020303-s001.pdf]

Online Supplementary Document
Panch et al. Artificial intelligence, machine learning and health systems

J Glob Health 2018;8:020303

References s21-s61

21 Jha S, Topol EJ. Adapting to artificial intelligence: radiologists and pathologists as information specialists. JAMA. 2016;316:2353-54.
22 Atun R. Health systems, systems thinking and innovation. Health Policy Plan. 2012;27(suppl_4):iv4-8.
23 Celi LA, Davidzon G, Johnson AE, Komorowski M, Marshall DC, Nair SS, et al. Bridging the health data divide. JMIR. 2016;18:e325.
24 Prince JD. Precision Medicine: An Introduction. Journal of Electronic Resources in Medical Libraries. 2017;14:120-9.
25 Rajkomar A, Oren E, Chen K, et al., 2018. Scalable and accurate deep learning for electronic health records. arXiv preprint arXiv:1801.07860.
26 d'Alessandro B, O'Neil C, LaGatta T. Conscientious classification: a data scientist's guide to discrimination-aware classification. Big Data. 2017;5:120-34.
27 Hodson H. Google knows your ills. New Scientist. 2016;230:22-23.
28 Parliament and Council of the European Union (2016). Regulation Regulation (EU) 2016/679 of the European Parliament and of the Council of 27 April 2016 on the protection of natural persons with regard to the processing of personal data and on the free movement of such data, and repealing Directive 95/46/EC (General Data Protection Regulation). Available: https://publications.europa.eu/en/publication-detail/-/publication/3e485e15-11bd-11e6-ba9a-01aa75ed71a1/language-en. Accessed: 20 March 2018.
29 Doshi-Velez F, Kortz M, Budish R, et al. Accountability of AI under the law: the role of explanation. arXiv preprint arXiv:1711.01134. 2017 Nov 3.
30 Trister AD, Buist DS, Lee CI. Will machine learning tip the balance in breast cancer screening?. JAMA Oncology. 2017;3:1463-4.
31 Fergus P, Hussain A, Al-Jumeily D, Huang DS, Bouguila N. Classification of caesarean section and normal vaginal deliveries using foetal heart rate signals and advanced machine learning algorithms. Biomed Eng Online. 2017;16:89.
32 Ambale-Venkatesh B, Wu CO, et al. Cardiovascular event prediction by machine learning: the multi-ethnic study of atherosclerosis. Circ Res. 2017;121:1092-101.
33 Poplin R, Varadarajan AV, Blumer K, Liu Y, McConnell MV, Corrado GS, et al. Prediction of cardiovascular risk factors from retinal fundus photographs via deep learning. Nature Biomedical Engineering 2018;2:158–64.
34 Berchialla P, Foltran F, Bigi R, Gregori D. Integrating stress-related ventricular functional and angiographic data in preventive cardiology: a unified approach implementing a Bayesian network J Eval Clin Pract. 2012;18:637-43.
35 Hu B, Dixon PC, Jacobs JV, Dennerlein JT, Schiffman JM. Machine learning algorithms based on signals from a single wearable inertial sensor can detect surface-and age-related differences in walking. J Biomech. 2018;71:37-42.

36 Katzman J, Shaham U, Bates J, Cloninger A, Jiang T, Kluger Y. DeepSurv: personalized treatment recommender system using a Cox proportional hazards deep neural network. BMC Medical Research Methodology (2018) 18:24.
37 Bejnordi BE, Veta M, van Diest PJ, et al. Diagnostic assessment of deep learning algorithms for detection of lymph node metastases in women with breast cancer. JAMA. 2017;318:2199-10.
38 Bychkov D, Linder N, Turkki R, et al. Deep learning based tissue analysis predicts outcome in colorectal cancer. Sci Rep. 2018;8:3395.
39 Han SS, Kim MS, Lim W, Park GH, Park I, Chang SE. Classification of the clinical images for benign and malignant cutaneous tumors using a deep learning algorithm. J Invest Dermatol. 2018;138:1529-38.
40 Han SS, Park GH, Lim W, Kim MS, Im Na J, Park I, et al. Deep neural networks show an equivalent and often superior performance to dermatologists in onychomycosis diagnosis: Automatic construction of onychomycosis datasets by region-based convolutional deep neural network. PloS one. 2018;13:e0191493.
41 Esteva A, Kuprel B, Novoa RA, Ko J, Swetter SM, Blau HM, Thrun S. Dermatologist-level classification of skin cancer with deep neural networks. Nature. 2017;542:115.
42 Yu KH, Zhang C, Berry GJ, Altman RB, Ré C, Rubin DL,et al. Predicting non-small cell lung cancer prognosis by fully automated microscopic pathology image features. Nat Commun. 2016;16:12474.
43 Ting DS, Cheung CY, Lim G, Tan GS, Quang ND, et al. Development and validation of a deep learning system for diabetic retinopathy and related eye diseases using retinal images from multiethnic populations with diabetes. JAMA. 2017;318:2211-23.
44 Burlina PM, Joshi N, Pekala M, Pacheco KD, Freund DE, Bressler NM. Automated grading of age-related macular degeneration from color fundus images using deep convolutional neural networks. JAMA Ophthalmol. 2017;135:1170-6.
45 Brisimi TS, Chen R, Mela T, Olshevsky A, Paschalidis IC, Shi W. Federated learning of predictive models from federated Electronic Health Records. Int J Med Inform. 2018;112:59-67.
46 Berikol GB, Yildiz O, Özcan İT. Diagnosis of acute coronary syndrome with a support vector machine. Journal of medical systems. 2016;40:84.
47 Inan OT, Pouyan MB, Javaid AQ, Dowling S, Etemadi M, Dorier A, et al. Novel wearable seismocardiography and machine learning algorithms can assess clinical status of heart failure patients. Circ Heart Fail. 2018;11:e004313.
48 Rajkomar A, Yim JW, Grumbach K, Parekh A. Weighting primary care patient panel size: A novel electronic health record-derived measure using machine learning. JMIR Med Inform. 2016;4:e29.
49 Chougrad H, Zouaki H, Alheyane O. Deep convolutional neural networks for breast cancer screening. Comput Methods Programs Biomed. 2018;157:19-30.
50 McCoy A, Das R. Reducing patient mortality, length of stay and readmissions through machine learning-based sepsis prediction in the emergency department, intensive care unit and hospital floor units. BMJ Open Qual. 2017;6:e000158.
51 Parreco JP, Hidalgo AE, Badilla AD, Ilyas O, Rattan R. Predicting central line-associated bloodstream infections and mortality using supervised machine learning. J Crit Care. 2018;45:156-62.

52 Miotto R, Li L, Kidd BA, Dudley JT. Deep patient: an unsupervised representation to predict the future of patients from the electronic health records. Sci Rep 2016;6:26094.
53 Horng S, Sontag DA, Halpern Y, Jernite Y, Shapiro NI, Nathanson LA. Creating an automated trigger for sepsis clinical decision support at emergency department triage using machine learning. PloS One. 2017;12:e0174708.
54 Forsyth AW, Barzilay R, Hughes KS, Lui D, Lorenz KA, Enzinger A5, et al. Machine learning methods to extract documentation of breast cancer symptoms from electronic health records. J Pain Symptom Manage. 2018;55:1492-99.
55 Blecker S, Katz SD, Horwitz LI, Kuperman G, Park H, Gold A, Sontag D. Comparison of approaches for heart failure case identification from electronic health record data. JAMA Cardiol. 2016;1:1014-20.
56 Gehrmann S, Dernoncourt F, Li Y, Carlson ET, Wu JT, Welt J, et al. Comparing deep learning and concept extraction based methods for patient phenotyping from clinical narratives. PloS One. 2018;13:e0192360.
57 Weng WH, Wagholikar KB, McCray AT, Szolovits P, Chueh HC. Medical subdomain classification of clinical notes using a machine learning-based natural language processing approach. BMC Med Inform Decis Mak. 2017;17:155.
58 Duarte F, Martins B, Pinto CS, Silva MJ. Deep neural models for ICD-10 coding of death certificates and autopsy reports in free-text. J Biomed Inform. 2018;80:64-77.
59 Hoogendoorn M, Berger T, Schulz A, Stolz T, Szolovits P. Predicting social anxiety treatment outcome based on therapeutic email conversations. IEEE Journal of Biomedical and Health iInformatics. 2017;21:1449-59.
60 Rumshisky A, Ghassemi M, Naumann T,et al. Predicting early psychiatric readmission with natural language processing of narrative discharge summaries. Translational Psychiatry. 2016;6:e921.
61 Razavian N, Blecker S, Schmidt AM, Smith-McLallen A, Nigam S, Sontag D. Population-level prediction of type 2 diabetes from claims data and analysis of risk factors. Big Data. 2015;3:277-87.
